# Identification of a plant extract that differentially inhibits harmful and beneficial skin-dwelling bacteria

**DOI:** 10.17912/micropub.biology.002208

**Published:** 2026-10-22

**Authors:** Geraldine Misquith, Ianna Burke, Jennifer Chandler

**Affiliations:** 1 Biology, West Chester University, West Chester, PA, United States; 2 Chemistry, West Chester University, West Chester, PA, United States

## Abstract

Many skin care products kill both harmful and beneficial skin bacteria. We tested four plant extracts to determine if any potentially harmful bacteria is targeted while preserving beneficial bacteria. Two bacterial species, *Staphylococcus aureus* and *Staphylococcus epidermidi* s, were exposed to four commonly used extracts: *Matricaria recutita* , *Cistus ladanifer* , *Pogostemon cablin* , and *Artemisia herba-alba* . *Matricaria recutita* did not impact either bacterium. *Pogostemon cablin* and *A. herba-alba* inhibited both bacteria equally, whereas *C. ladanifer* strongly inhibited *S. aureus* while having a much weaker effect on *S. epidermidis* . Results suggest *C. ladanifer* may be a promising, safe natural ingredient for skin care products.

**Figure 1. Experimental design, representative plates, and graphical display of the interactive effect of extract type and bacterial species on zone of inhibition:** Figure 1A depicts the factorial design comparing inhibition among eight combinations of plant extract type and bacterial species. Figure 1B shows representative plates illustrating disk placement and zone of inhibition in both *S. aureus* and *S. epidermidis* exposed to discs soaked in sterile H _2_ O ***,*** *M. recutita, C. ladanifer* , *A. herba-alba* Asso, and *P. cablin.* Figure 1C graphs mean zone of inhibition (mm ^2^ ) as a function of plant extract type and bacterial species. Bars representing the mean zone of bacterial inhibition (mm ^2^ ) of each treatment combination share the same letter if their means do not differ (p>0.05). Error bars represent 95% confidence intervals.

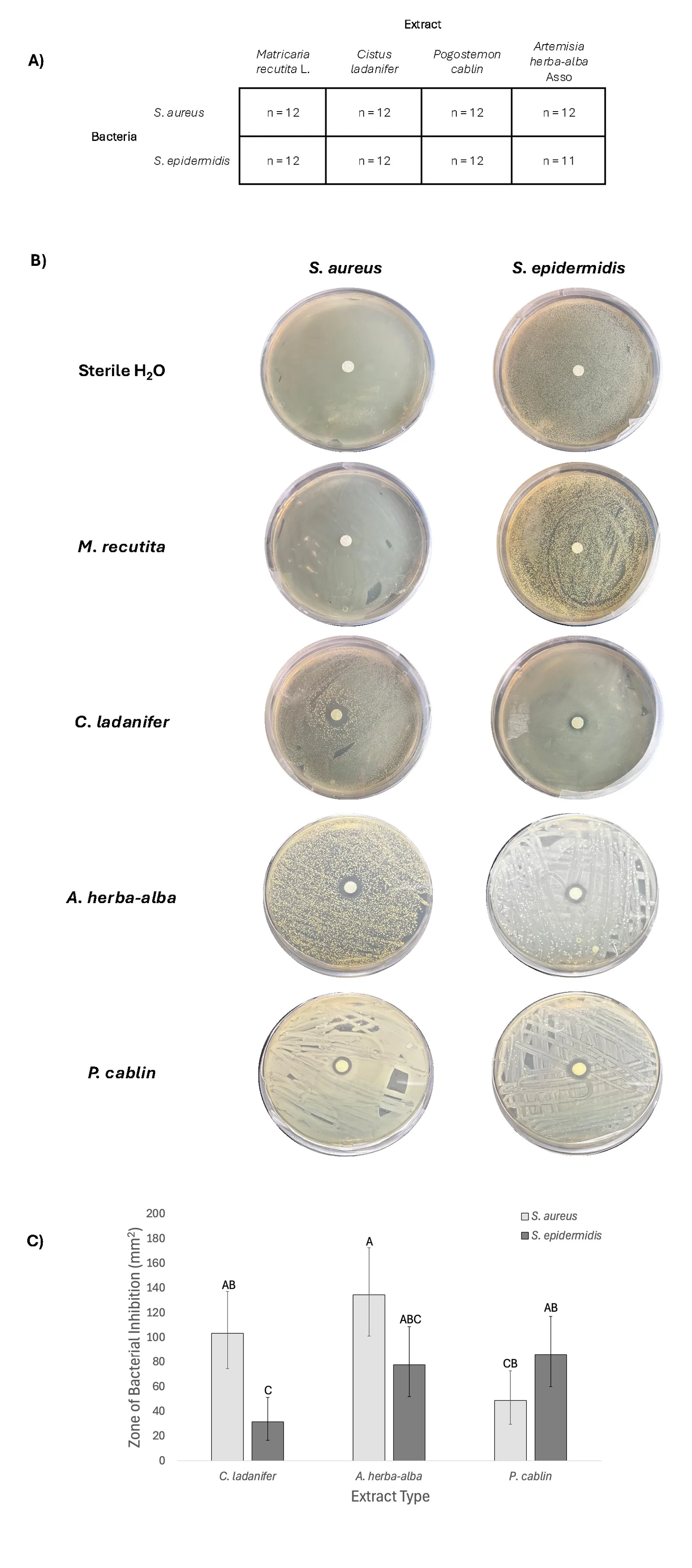



## Description

Within a single generation, social media and celebrity culture have driven women, men, and even children to strive for unrealistic physical “perfection.” It is no surprise that during this same period, the popularity of personal care products and cosmetics promising youthful glows and enhanced beauty has skyrocketed among all demographics, but particularly among young people (Alnuqaydan, 2024). A reported 90% of individuals use some form of personal care product every day (Alnuqaydan, 2024). Nevertheless, there is a dark side to the personal care industry of which most of the users are not aware; repeated exposure to many of the synthetic compounds present in self-care products may increase their risk of health complications compared to users of natural products (Alnuqaydan, 2024). Moreover, cumulative combined exposure to harmful chemicals found in many skin care products produce non-additive risks beyond what might be elicited from any single compound (Alnuqaydan, 2024). Skin creams and lotions touted to even skin tone, reduce acne, smooth wrinkles, and brighten dullness often contain compounds such as butylated hydroxyanisole (Kaushik et al. 2023), butylated hydroxytoluene (Balwierz et al., 2023), diazolidinyl urea (de Groot and Flyvholm, 2020), parabens and propylparabens (Alaba et al., 2022; Downs et al., 2023) and polyethylene glycols (Caballero et al., 2021) that can cause skin irritation, allergic reaction, and at their worst, hormone and endocrine disruption and cancer concerns (Alnuqaydan, 2024). Clearly more natural, and importantly safer, dermatological products are needed.

Many skin care products marketed largely to teenagers and young adults use harsh chemicals to sanitize the skin’s surface and aid in clearing acne. These products work in part by killing bacteria on the skin’s surface, however, many of these products fail to discriminate between the harmful skin-dwelling bacteria that cause irritation and the beneficial skin bacteria that stabilize the skin barrier. It seems that natural, plant-based dermatological alternatives are needed that discriminate between the type of bacteria they kill. The purpose of our work was to identify a plant extract that is gentle and safe for the skin, but that inhibits growth of harmful bacteria while simultaneously preserving beneficial skin bacteria.

Two species of bacteria were selected for this experiment. The first was *Staphylococcus aureus* , a gram-positive, typically commensal bacteria that colonizes the skin and/or nasal passage of around 30% of the population (Ryu et al., 2014). Harmful infections caused by *S. aureus* can occur if introduced into the bloodstream or through skin and wound infections, and can range from minor skin infections to abscesses, endocarditis, and sepsis (Ryu et al., 2014). The second was *Staphylococcus epidermidis* , a gram-positive bacterium that is widely considered a beneficial skin bacteria that helps maintain the skin barrier, ultimately preventing water loss and protecting against pathogens (Zheng et al., 2022; Qi et al., 2025), though Brown and Horswill (2020) provide a more nuanced interpretation. Four commercial plant extracts were chosen for this study due to their widespread availability, their popularity as anecdotal home remedies, and their ethnobotanical legacies: chamomile ( *Matricaria recutita* L.), cistus ( *Cistus ladanifer* ), white wormwood ( *Artemisia herba-alba* Asso), and patchouli ( *Pogostemon cablin* ). Twelve replicates of each bacterium and extract treatment combination were established in a 2x4 factorial design ( Fig. 1A ) though one replicate in the white wormwood + *S. epidermidis* combination was discarded due to handler errors. Additional replicates of both *S. aureus* and S *. epidermidis* were plated with sterile water to serve as a negative control.

Negative controls confirmed that the two bacterial cultures we used grew as expected when not exposed to potential inhibitory extracts ( Fig. 1B ). *Matricaria recutita* was wholly ineffective against both *S. aureus* and *S. epidermidis* , with no observable zone of inhibition in any replicate ( Fig. 1B ). As such, treatment combinations including *M. recutita* were removed from statistical analyses. A two-way ANOVA using the remaining three extracts indicates that there is a differential response of inhibition based on bacterial species and extract type ( Fig. 1C ; F _BxE_ = 10.570, p = 0.001), and while the remaining three extract types inhibited bacterial growth, generally, the patterns of inhibition differed based on the species of bacteria. There was no difference in the zone of inhibition between plates inoculated with *S. aureus* and *S. epidermidis* when exposed to *A. herba-alba* , and the same was true in exposure to *P. cablin* . Both extracts suppressed the growth of each of the two bacteria similarly. *Artemisia herba-alba* Asso was more effective at inhibiting growth of *S. aureus* than *P. cablin* , but their effect on *S. epidermidis* did not differ. Interestingly, *C. ladanifer* was unique among the chosen extracts in that it inhibited growth of the potentially harmful bacteria, *S. aureus* , to a much greater extent than it did the beneficial skin bacteria, *S. epidermidis* ( Fig. 1B, C), which is the pattern of response we were seeking.

Other studies have demonstrated and described the effectiveness of *C. ladanifer* as an antimicrobial agent and have ascribed efficacy to the major secondary metabolites found therein, including viridiflorol, *trans* -pinocarveol, bornyl acetate, and ledol (Yashphe et al *.,* 1979; Yashphe et al *.,* 1987; Bertella et al., 2018; El Karkouri et al., 2021). These secondary metabolites act primarily by disrupting the bacterial membrane and inhibiting enzymes and metabolic pathways, among other mechanisms. What is less clear is how *C. ladanifer* differentially affects these two bacterial species; further work teasing apart the mechanisms behind this impact are needed. Additionally, prior work has shown that the chemical properties of this plant vary based on growing location, harvest time, and extraction type (El Karkouri et al *.* , 2021; Kielar et al., 2025), and it is likely that efficacy will vary based on these factors. Additional work should focus on how these differences may impact the ability of *C. ladanifer* to differentially inhibit growth of different types of bacteria.

Nevertheless, the consensus that *Cistus ladanifer* is considered safe for human use (Kielar et al., 2025), combined with its demonstrated ability to selectively inhibit a potentially harmful bacterium while showing less activity against a beneficial skin bacterium, suggests that *C. ladanifer* may have potential promise as a safe and natural alternative to synthetic dermatological products.

## Methods

**Preparation of Culture Plates**

Pure cultures of *S. aureus* and *S. epidermidis* were grown overnight at 37 ^o^ C on non-selective tryptic soy agar (TSA) media plates to avoid atypical variants. A single colony of each culture was used to prepare a suspension medium using 5ml tryptic soy broth tubes overnight. Suspension density was adjusted to match a 0.5 McFarland turbidity standard. The prepared inoculants were used within 1hr to ensure viability. A swab method was used to inoculate Mueller-Hinton agar plates with each of the two cultures. The prepared, inoculated plates were sealed and stored in a 4 ^o^ C refrigerator for one week prior to experimentation to accommodate breaks in the academic calendar. This one-week delay in experimentation ensured that researchers were on site to monitor the experiment daily.

**Extract Application, Incubation, and Measurement of Inhibition**

Plates were removed from cold storage after one week, and sterile paper discs (28.27mm ^2^ ) were soaked in one of four extracts or sterile water. Sterilized forceps were used to place one disc in the center of each inoculated plate in a 2-way factorial design ( Fig. 1A ). Plates were incubated in a room temperature cabinet (approximately 21 ^o^ C) for one week. The zone of inhibition (mm ^2^ ) was calculated for each plate as the area of bacterial inhibition surrounding the disc, minus the area of the disc itself ( Fig. 1B ).

**Statistical Analyses**

*Matricaria recutita* did not have any inhibitory effect on *S. aureus* and *S. epidermidis* and was removed from the dataset to avoid an overabundance of zeros in the data. A square root data transformation was required to meet the assumption of normality of residuals (W = 0.985, p = 0.569). Homogeneity of variance was not met; however, ANOVA is robust to heteroscedasticity when designs are nearly balanced, which was the case in this study ( Fig. 1A ). As such, we proceeded with a two-way ANOVA with the response variable being the measured zone of inhibition (mm ^2^ ), the first explanatory variable being the plant extract type, and the second explanatory variable being species of bacteria. A Tukey-HSD post hoc analysis was used to determine differences among means in the significant interaction term. Means and confidence intervals were back-transformed for presentation.

## Reagents

**Table:** 

**Name**	**Strain/Description**	**Concentration**	**Supplier**	**Catalog Number**
*Staphylococcus aureus* subsp. *aureus*	NCTC 8532 [IAM 12544, R. Hugh 2605]	-	ATCC	Cat. No. 12600
*Staphylococcus epidermidis*	Fussel [NCTC 11047, R. Hugh 2466]	-	ATCC	Cat. No. 14990
*Artemisia herba-alba*	Steam distilled, Lot 14	100% pure essential oil - undiluted	Eden Botanicals	SKU 78-3
*Cistus ladanifer*	Steam distilled, Lot 7	100% pure essential oil - undiluted	Eden Botanicals	SKU 222-030
*Pogostemon cablin*	Steam distilled, Lot 21	100% pure essential oil - undiluted	Eden Botanicals	SKU 760-9
*Matricaria recutita* L.	Steam distilled, Lot 21	100% pure hydrosol (1% volatiles, 99% H _2_ O	Eden Botanicals	SKU 1548-9
